# Therapeutic Potential of 6-Gingerol in Prevention of Colon Cancer Induced by Azoxymethane through the Modulation of Antioxidant Potential and Inflammation

**DOI:** 10.3390/cimb44120424

**Published:** 2022-12-08

**Authors:** Abdulaziz A. Aloliqi

**Affiliations:** Department of Medical Biotechnology, College of Applied Medical Sciences, Qassim University, Buraydah 51542, Saudi Arabia; aaalieky@qu.edu.sa

**Keywords:** 6-gingerol, azoxymethane, colon cancer, inflammation, antioxidant potential

## Abstract

A polyphenolic component of ginger, 6-gingerol, is widely reported to possess antioxidant, anti-inflammatory and anticancer activities. In the current study, it was aimed to investigate the anticancer effects of 6-gingerol (6-Gin) on azoxymethane (AOM)-induced colon cancer in rats. The results reveal that 6-Gin treatment significantly improves the antioxidant status disturbed by AOM intoxication. The 6-Gin treatment animal group showed enhanced activity of catalase (CAT) (46.6 ± 6.4 vs. 23.3 ± 4.3 U/mg protein), superoxide dismutase (SOD) (81.3 ± 7.6 vs. 60.4 ± 3.5 U/mg protein) and glutathione-S-transferase (GST) (90.3 ± 9.4 vs. 53.8 ± 10 mU/mg protein) (*p* < 0.05) as compared to the disease control group. Furthermore, the results reveal that AOM significantly enhances the inflammatory response and 6-gingerol potentially attenuates this response, estimated by markers, such as tumor necrosis factor-α (TNF-α) (1346 ± 67 vs. 1023 ± 58 pg/g), C-reactive protein (CRP) (1.12 ± 0.08 vs. 0.92 ± 0.7 ng/mL) and interleukin-6 (IL-6) (945 ± 67 vs. 653 ± 33 pg/g). In addition, the lipid peroxidation estimated in terms of malondialdehyde (MDA) provoked by AOM exposure is significantly reduced by 6-gingerol treatment (167 ± 7.5 vs. 128.3 nmol/g). Furthermore, 6-gingerol significantly maintains the colon tissue architecture disturbed by the AOM treatment. Loss of tumor suppressor protein, phosphatase and tensin homolog (PTEN) expression was noticed in the AOM treated group, whereas in the animals treated with 6-gingerol, the positivity of PTEN expression was high. In conclusion, the current findings advocate the health-promoting effects of 6-gingerol on colon cancer, which might be due to its antioxidant and anti-inflammatory potential.

## 1. Introduction

Colon cancer is a one of the leading causes of cancer deaths in both men and women in most countries, including Saudi Arabia. Globally, it is the fourth most common cause of mortality due to cancer [1]. It is also one of the most preventable cancers, as several risk factors, such as eating red and processed meat, sedentarism, alcoholism, smoking, etc., can control this disease [2]. The carcinogenesis of colon cancer is related to both genetic and environmental factors. Different types of chemicals, such as benzopyrene [B(a)P], diethyl nitrosamine (DEN) and azoxymethane (AOM), are routinely used in different industrial and research sectors, and are the main culprit in colon-associated pathogenesis, including cancer [3].

The AOM-induced colon cancer in animal models was used to study the different mechanisms of this carcinogen to understand, in parallel, the role of this carcinogen for human sporadic colorectal cancer [4]. The AOM exposure leads to colorectal epithelial pathogenesis from minor lesions to adenoma and malignant adenocarcinoma [5]. The in vivo AOM metabolism promotes chromosomal damage and DNA mutations by changing the nucleotide sequence [6].

The current mode of treatment, including surgery, chemotherapy and radiotherapy, may be effective, but such types of treatments are expensive and are toxic to normal cells [7,8]. Therefore, safe and cheaper modes of treatment are needed to overcome the complications caused by current treatment modules. In this regard, the treatment based on natural products or their active compounds plays a significant role in the management of colorectal cancer development and progression [9,10]. Ginger (*Zingiber officinale*) has been used as a traditional medicine for a long time [11]. The ginger rhizome constitutes many phenolic compounds, including zingerone, 6-paradol, 6-shago, and 6-gingerol [12].

One of the major aromatic polyphenolic compounds of ginger is 6-Ginerol (1-[49-hydroxy-39-methoxyphenyl]-5-hydroxy-3-decanone), which has potent and antioxidant and anti-inflammatory activity that defends cells from oxidative stress and cell death [13]. This pharmacologically active compound possesses substantial antimutagenic and anticarcinogenic activities [14]. An abundance of mounting evidence supports that 6-gingerol is potentially effective in suppressing the transformation, hyperproliferation and inflammatory changes that promote carcinogenesis, angiogenesis and metastasis [15,16,17]. Despite the awareness of 6-gingerol therapeutic potential against several cancers, the exact molecular mechanism underlying the anticancer role of 6-gingerol is not fully clear.

The aim of the present study was to explore the effect of 6-gingerol against long-term administration of AOM-induced disturbances in the colons of rats. The therapeutic potential of 6-gingerol was evaluated by assaying its antioxidant, anti-inflammatory, lipid peroxidation and histopathological alteration.

## 2. Materials and Methods

### 2.1. Reagents and Chemicals

The present study used azoxymethane, obtained from Sigma Aldrich, Co., St. Louis, MO, USA. The antioxidant enzyme ELISA kits (GST, SOD and CAT) were purchased from Abcam, Cambridge, UK. The inflammatory markers (IL-6, TNF-α, CRP) and lipid peroxidation detection ELISA kit (MDA) were also obtained from Abcam, Cambridge, UK. Primary monoclonal antibodies against PTEN were purchased from Abcam, Cambridge UK. All other reagents and chemicals were obtained from commercial sources and were guaranteed to be of the highest available grade.

### 2.2. Animals

In this study, male albino Wister rats (180–200 g) were used, purchased from the animal house of King Saud University, Riyadh, Saudi Arabia. The standard environmental and food conditions were provided to all the animals i.e., 23 ± 1 °C, 55 ± 5% humidity and 12 h light/12 h dark cycle with free food and water access. The animals were acclimatized for one week to get relief from transportation stress and to habituate to a new environment. The CAMS ethical committee, Qassim University, approved the use of the animal model for this study (Grant no. 10082-cams1-2020-1-1w). The guidelines of the NIH, USA, were followed for the care and experimental use of animals.

### 2.3. Animals Groups

All the animals were randomly and double blindly assigned to four groups to avoid experimental bias (each group containing 8 rats) (Table 1). The azoxymethane (AOM) and 6-gingerol were dissolved separately in normal saline. The rats were randomly divided into four groups (*n* = 8 in each group) and the treatment plan (Table 1) was continued for 32 weeks. Then, 2 doses/week were giving to each rat in groups number 2, 3 and 4.

### 2.4. Tissue Collection and Measurement of Antioxidant and Anti-Inflammatory Markers

At the end of the treatment plan of 32 weeks, all animals were anesthetized by urethane (1.5 g/kg i.p.). The colon samples were collected and washed in normal saline for each rat and treated individually for studying the colon samples (one by one). Each colon sample was divided into two portions. One portion was collected in phosphate buffer saline (pH 7.0) to analyse the antioxidant and anti-inflammatory markers. The colon tissues were homogenized with 50 mM of phosphate buffer and the supernatants were collected for the estimation of different parameters of antioxidant and anti-inflammatory marker parameters. The homogenate was centrifuged at 10,000 rpm for 15 min at 4 °C. The supernatants were isolated for the estimation of antioxidant and anti-inflammatory markers.

### 2.5. Histopathological Analysis

The other section of colon from each rat was collected for histopathological studies. Neutral buffered saline (10%) was used to fix all the colorectal tissue samples, graded alcohol was used as the dehydrating agent, xylene was used to clear the tissues, paraffin wax was used as an embedding medium and the tissues were sectioned to a thickness of 5 μm using a microtome (Leica, Heidelberg, Germany). The sections were stained with hematoxylin and eosin for histopathological studies. Finally, the sections were observed under Leica microscope (Leica, Heidelberg, Germany) and photographs were captured.

### 2.6. Expressional Evaluation of PTEN Protein through Immunohistochemical Analysis

In order to examine the expression pattern of PTEN protein in the colon tissue, thin sections (5 µm sections) were used to evaluate the expression pattern using immunohistochemical staining as previously described [20,21]. The scoring for PTEN protein expressions were examined by independent pathologists in a blinded means. The PTEN scoring uses a method to measure PTEN expression in colon tissue. When PTEN expression was less than 5%, it was considered negative. Expression levels from 5–15% were considered as 1, 15–13% as 2, 30–50% as 3 and more than 50% considered as 4. The expression of PTEN protein was examined in a semi-quantitative manner, in which the staining intensity was multiplied by the % of positive cells.

### 2.7. Statistical Analysis

The data obtained was expressed in mean ± SD. One-way analysis of variance (ANOVA) was used to calculate the significance of differences among various groups. Statistical package for social sciences (SPSS) software was used to perform the statistical analysis. The significant difference (*p* < 0.05) was measured between the different experimental groups.

## 3. Results

### 3.1. Effect of 6-Gingerol Treatment on Azoxymethane-Induced Colon Cancer through the Investigation of Antioxidant Enzyme Levels

**Figure 1:**
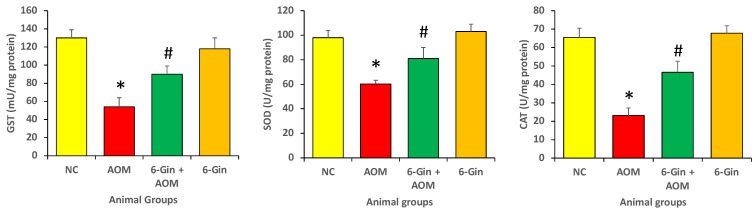
The protective effect of 6-Gingerol on the azoxymethane-induced colon carcinogenesis through enhancement of antioxidant enzyme levels. Data are represented as ± standard error of mean (SEM). The different groups are NC as normal control, AOM as azoxymethane treatment group (disease control), 6-Gin + AOM as 6-gingerol treatment to disease control and 6-Gin as only 6-gingerol treatment group. The * indicates significant difference (*p* < 0.05) between disease control vs. normal control and # indicates significant difference (*p* < 0.05) between disease control and disease control with treatment.

The level of antioxidant enzymes, such as glutathione-S-transferase (GST), superoxide dismutase (SOD) and catalase (CAT), were investigated in different animal groups, and it was observed that the level of these antioxidant enzymes markedly decreased in colon cancer as compared to the normal control. Treatment with 6-gingerol enhanced the decreased GST, SOD and catalase when compared to the AMO-only treated group (*p* < 0.05). (Figure 1). Moreover, the level of antioxidant enzymes was measured in the control and 6-gingerol groups, and it was very similar; the differences in the enzyme levels in these two groups was statistically insignificant. As compared with the 6-gingerol group, the antioxidant enzyme levels were low in the 6-gingerol plus AOM treatment group (Figure 1). The 6-Gin treatment group showed enhanced activity of catalase (CAT) (46.6 ± 6.4 vs. 23.3 ± 4.3 U/mg protein), superoxide dismutase (SOD) (81.3 ± 7.6 vs. 60.4 ± 3.5 U/mg protein) and glutathione-S-transferase (GST) (90.3 ± 9.4 vs. 53.8 ± 10 mU/mg protein) (*p* < 0.05), as compared to the disease control group. 

### 3.2. Effect of 6-Gingerol Treatment on Azoxymethane-Induced Colon Cancer through the Investigation of Inflammatory Protein Marker Levels

**Figure 2:**
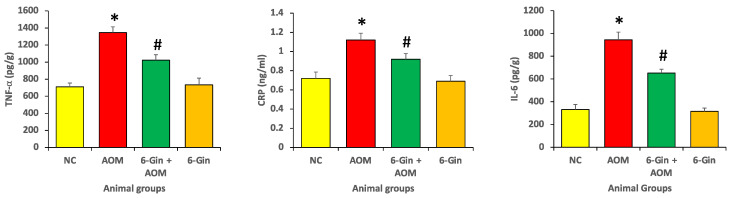
The anti-inflammatory effect of 6-Gingerol on the azoxymethane-induced inflammatory cytokine level. Data are represented as ± standard error of mean (SEM). The different groups are NC as normal control, AOM as azoxymethane treatment group (disease control), 6-Gin + AOM as 6-gingerol treatment to disease control and 6-Gin as only 6-gingerol treatment group. The * indicates significant difference (*p* < 0.05) between disease control vs. normal control and # indicates significant difference (*p* < 0.05) between disease control and disease control with treatment.

The level of inflammatory protein markers, including interleukin-6 (IL-6), tumor necrosis factor-alpha (TNF-α) and C-reactive protein (CRP), were investigated in different animal groups, and it was observed that the level of these inflammatory protein markers markedly increased in the colon cancer disease control groups as compared to the normal control and 6-gingerol treatment groups (Figure 2).

Moreover, the level of inflammatory markers TNF-α, CRP and IL-6 were measured in the control and 6-gingerol groups, and it was very similar; the differences were statistically insignificant. As compared with the 6-gingerol group, the inflammatory markers were high in the 6-gingerol plus AOM treatment group (Figure 2). The results reveal that AOM significantly enhances the inflammatory response and 6-gingerol potentially attenuates this response, estimated by markers including tumor necrosis factor-α (TNF-α) (1346 ± 67 vs. 1023 ± 58 pg/g), C-reactive protein (CRP) (1.12 ± 0.08 vs. 0.92 ± 0.7 ng/ml) and interleukin-6 (IL-6) (945 ± 67 vs. 653 ± 33 pg/g).

### 3.3. Effect of 6-Gingerol Treatment on Azoxymethane-Induced Colon Cancer through the Investigation of Lipid Peroxidation

**Figure 3:**
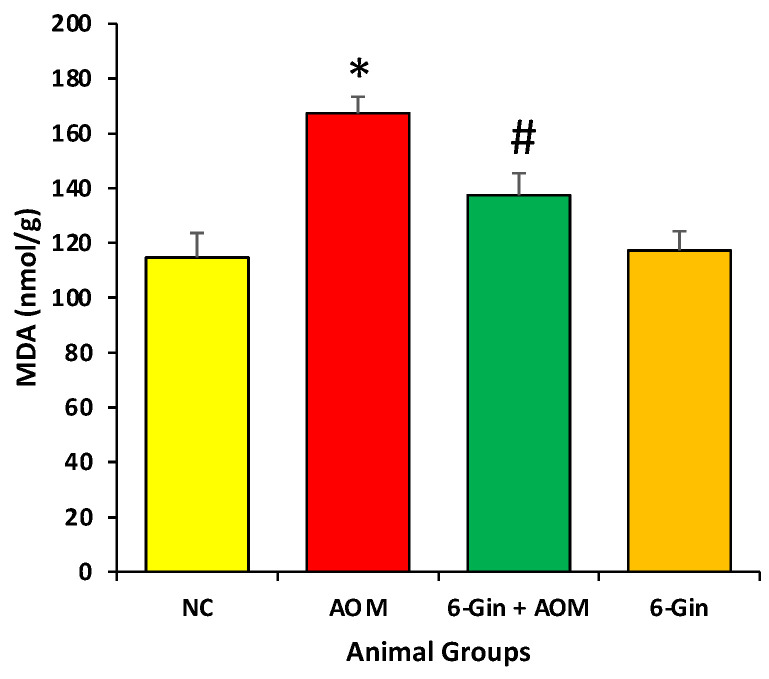
The protective effect of 6-Gingerol on the azoxymethane-induced lipid peroxidation level. Data are represented as ± standard error of mean (SEM). The different groups are NC as normal control, AOM as azoxymethane treatment group (disease control), 6-Gin + AOM as 6-gingerol treatment to disease control and 6-Gin as only 6-gingerol treatment group. The * indicates significant difference (*p* < 0.05) between disease control vs. normal control and # indicates significant difference (*p* < 0.05) between disease control and disease control with treatment.

Malondialdehyde (MDA), the main indicator of lipid peroxidation, was measured in the colon tissues samples by using thiobarbituric acid reactive substances (TBARS). The concentration of the MDA level was significantly increased in the AOM-treated colon cancer group, as compared to the control groups (*p* < 0.05). In the 6-gingerol treated groups, the MDA level was significantly reduced, as compared to the AOM-caused colon cancer group (*p* < 0.05). These findings demonstrate that AOM promotes potential oxidative damage in colon and 6-gingerol attenuates these changes (Figure 3). Moreover, the level of MDA was measured in the control and 6-gingerol groups and it was very similar; the differences were statistically insignificant. As compared with the 6-gingerol group, the MDA level was high in the 6-gingerol plus AOM treatment group (Figure 3). The lipid peroxidation estimated in terms of malondialdehyde (MDA) provoked by AOM exposure is significantly reduced by the 6-gingerol treatment (167 ± 7.5 vs. 128.3 nmol/g).

### 3.4. Role of 6-Gingerol on the Maintenance of Colon Tissue Architecture

**Figure 4:**
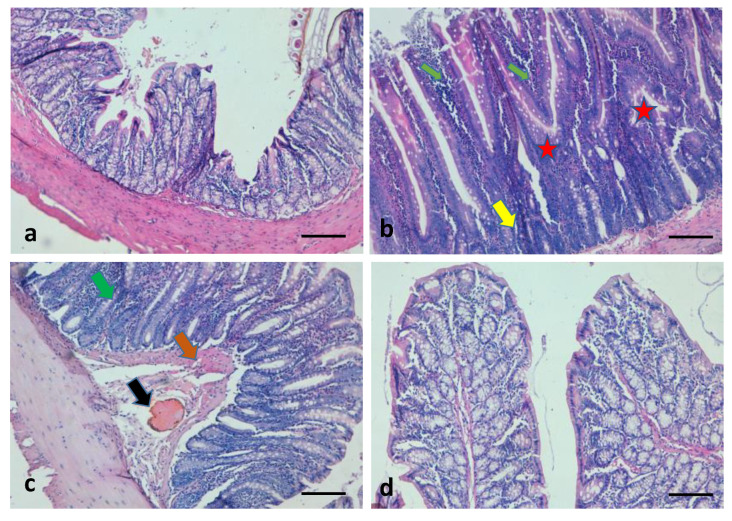
The colon protective role of 6-gingerol in different treatment groups. (**a**) control group showing normal architecture of the colon tissue, (**b**) AOM treated colon cancer group. The mucosal glands are irregular in size and shape (red star), hyperchromatic with severe aggregation of inflammatory cells (green arrow) and alteration of normal structure (yellow arrow). (**c**) AOM plus 6-gingerol group, colon cancer not found; however, inflammation (green color), congestion (black color) and fibrosis are seen (reddish orange), (**d**) 6-gingerol only treated group, showing normal architecture of colon tissue, (*n* = 8 animals/group) (Scale bar = 100 μm).

The colon tissue of the control group animals showed normal architecture. The AOM-treated group showed development of cancer in six out of eight rats; the other two rats generated severe inflammation congestion fibrosis. In the 6-Gingeral plus AOM treated group, not only was colon cancer observed, but inflammation, hemorrhage and fibrosis were seen, as well. However, these findings suggest that 6-gingerol has a colon protective role through the maintenance of colon tissue architecture (Figure 4).

### 3.5. The Role of 6-Gingerol on PTEN Protein Expression

**Figure 5:**
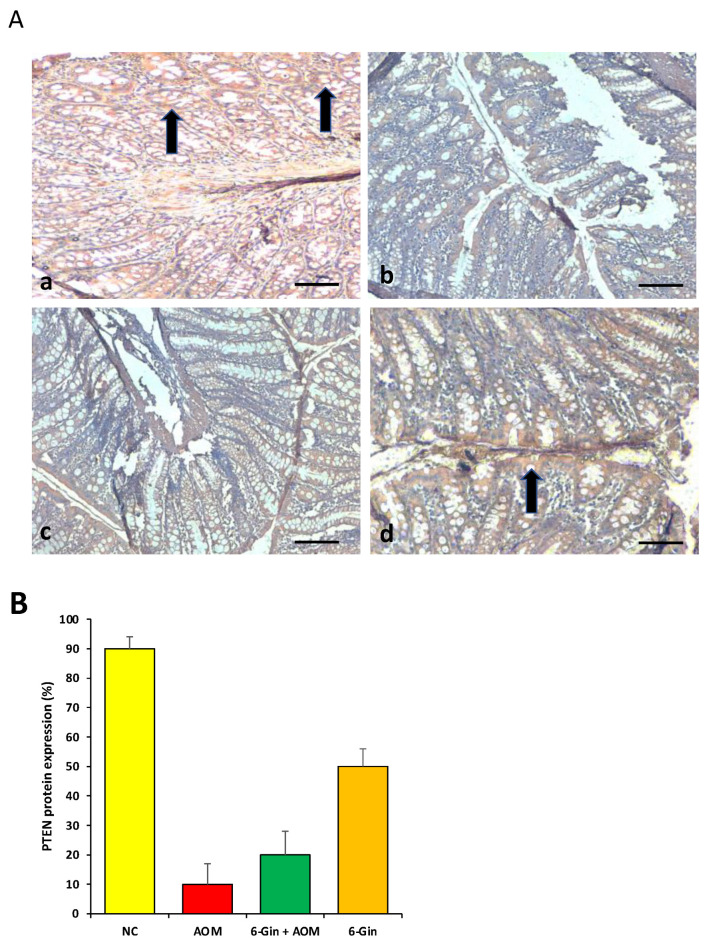
(**A**) PTEN protein expression analysis. (**a**) The control group showed high expression of PTEN (black arrow); (**b**) the group treated with AOM only displayed a loss of PTEN protein expression, (**c**) the expression of this marker protein was reduced in animals co-treated with both AOM and 6-gingerol together, but the positivity was less than AOM only treated group, (**d**) the animals treated with only 6-gingerol showed high expression of PTEN protein (*n* = 8 animals/group) (Scale bar = 100 μm). (**B**) Percentage of PTEN protein expression in different animal groups.

PTEN protein is a tumor suppressor protein, and it is evident, as its expression is high in normal tissue, whereas a loss of PTEN protein expression is noticed in various types of tumors. AOM is a proven colon carcinogen, and this carcinogen alters the various cell signalling pathways, including PTEN gene expression. In the current study, a loss of PTEN protein expression in the colon cancer tissue was noticed. In the 6-gingerol plus AOM treatment group, a loss of PTEN protein was also observed, but the expression pattern of the PTEN protein was significantly high, as compared to the colon cancer group (Figure 5). This finding reveals that 6-gingerol plays an important role in the modulation of the cell signaling pathway. In the 6-gingerol only treated group, PTEN protein expression was high, which indicates that 6-gingerol does not have any adverse effect on the PTEN gene activity.

## 4. Discussion

Colon cancer is a leading cause of cancer-related deaths worldwide, that is significantly preventable by changes in life-style [22]. The consumption of some common edible phytochemicals found in some medicinal plants and ginger has gained considerable attention as a promising way for reducing the incidence of different cancers, including colorectal cancer [23].

The rhizome of ginger is an ingredient of daily diets in many countries and is an active ingredient in many traditional systems of herbal medicines, like Oriental medicine and Ayurveda, for managing many ailments, including indigestion and other gastrointestinal disorders.

In addition to different constituents of ginger, the therapeutic role of 6-gingerol has been evaluated for its antioxidant, anti-inflammatory, antibacterial and antitumor properties [24,25,26]. Other studies also support the anticancer properties of such ginger phenolics against different cancers. The use of 6-gingerol has been found to reduce the growth of different murine tumors, such as colon carcinoma, renal cell carcinomas and melanomas, by augmenting the infiltrations of tumor-infiltrating lymphocytes [27].

The antioxidative enzymes including CAT, SOD and GPx protect the cells against oxidative damage, as such enzymes convert ROS into stable products [28]. 6-gingerol has been described to possess a significant antioxidant potential, and this feature has been demonstrated to perform a significant role in the initiation of antioxidation protective reactions. Thus, these results specify that the extraordinary antioxidant potential of 6-gingerol might contribute to its therapeutic efficacy, as it displays that its free radical scavenging ability may play a role in protection from oxidative damage caused by free ROS. This study reveals that AOM administration leads to a significant decrease in the level of antioxidant enzymes, including GST, GSH and SOD, in the colon tissue of rats, in comparison to the control animals. However, treatment of the rats with 6-gingerol after the AOM administration noticeably enhanced the activities of colon CAT, GST and SOD activities. Thus, these results provide evidence that 6-gingerol possesses a remarkable protective role against oxidative stress by increasing the activities of various antioxidant enzymes. A study based on colitis reported that 6-Gingerol powerfully prevented colonic oxidative damage through increasing the activities of glutathione content and antioxidant enzymes, and decreased the malondialdehyde levels and hydrogen peroxide, and improved the colonic alterations [29].

Some recent observations have demonstrated that inflammation is usually implicated in most organ damage and cancer ranging from the initial to the late stage [30]. Remarkably, the enhanced expression of pro-inflammatory cytokines leads to elevated of ROS and, hence, oxidative stress, resulting in cell damage [31,32]. The pro-inflammatory cytokines are reported to be enhanced in most cancers, which is considered a significant hallmark of the pathogenic state. Different pro-inflammatory markers, like TNF-α and IL-6, contribute to the pathogenesis of some organ damages and cancer. Interleukin 6 (IL-6) is rapidly produced during different infections and tissue injuries, and attributes response to host defense through the stimulation of acute phase responses, haematopoiesis and immune reactions [33].

In different studies, 6-gingerol mechanisms on colon cancer cell proliferation and angiogenesis were studied [1,13,16]. The mechanism of 6-gingerol in SW480 colon cancer cell proliferation was evaluated [1]. 6-gingerol significantly decreased colon cancer cells proliferation by inhabiting PMA-induced activation of MAP kinase and partially decreased the phosphorylation of p38-MAPK. Additionally, 6-gingerol inhibited the transcriptional binding of the transcription factor AP-1, which is a well-known transcription factor of promoting cell proliferation and survival. It significantly induced apoptosis in colon cancer cell line SW480 compared to normal cell lines by activating procaspase-8, procaspase-9 and procaspase-3 to their active fragments. The mechanism of 6-gingerol was also studied in colon cancer cell line LoVo cells [13]. 6-gingerol significantly decreased in LoVo cells proliferation by G2/M arrest. Therefore, LoVo cells treatment with 6-gingerol inhibited the level of CDK1, cyclin A and cyclin; however, the level of p27Kip1 and p21Cip1 were increased. 6-gingerol also played an anti-angiogenesis role in colon cancer cells by inhibiting VEGF, leading to reduction of endothelial cells and blocking capillary formation [16]. Therefore, 6-gingerol may inhibit the AOM induction of colon carcinogenesis in rats through one of these mechanisms.

In the current study, inflammatory marker levels were found to be pointedly increased in AOM-induced colon cancer rats, as compared to the control group. The treatment with 6-gingerol meaningfully decreased the inflammatory marker levels. Another recent study was in an accordance with the current study, as it was reported that 6-gingerol acts as an anti-inflammatory through the reduction of inflammatory markers [19,26]. In one study, it was demonstrated that the anti-inflammatory and antitumorigenic activities of the natural compound curcumin were shown in various mouse models of colon cancer. The administration of a 2% curcumin diet decreased the total number of intestinal polyps by 75% and reduced IL-1β, IL-6 and TNF-α protein expression [34].

A pioneer study was performed based on ginger *Echinacea anngustifolia* extract to investigate the inflammation and chronic pain in non-steroidal anti-inflammatory drugs (NSAIDs) poor responders. This finding advocates the use of this extract supplementation as useful in the treatment of knee osteoarthritis [35].

AOM administration led to colon cancer development and altered the colon tissue architecture. In the treatment group of 6-gingerol, colon cancer was not observed but severe inflammation, congestion and fibrosis was noticed. These findings indicate that 6-gingerol has a chemo-preventive role through the inhibition of colon cancer development.

Another finding reported that *Zingiber officinale* extract and 6-gingerol deliver defense by preventing oxidative degradation of a biological and maintained tissue architecture [36]. The expression of PTEN protein was evaluated in different groups and it was observed that a loss of PTEN protein expression occurred in the AOM-treated group/colon cancer. Moreover, the expression pattern of PTEN protein was high in the AOM and 6-gingerol treatment group, as compared to the AOM treatment group. Previous reports based on colon cancer reported that no PTEN expression was seen in some cancerous samples. PTEN expression was positively detected in 67.2% of colorectal cancer tissues and all of the adjacent non-cancerous samples were 100% positive [37].

Overall, these findings advocate that 6-gingerol attenuates AOM-induced colon carcinogenesis, probably through inhibiting oxidative stress, inflammation and maintenance of colon tissue architecture and PTEN protein expression. Thus, 6-gingerol may be an alternative therapy for improving the outcomes of AOM-induced colon carcinogenesis.

## 5. Conclusions

The results of the current study demonstrate that AOM induces a significant oxidative stress, enhances the inflammatory response and causes destructive changes in the colon tissue architecture. 6-gingerol has a significant potential to attenuate the oxidative stress and reduces the inflammatory response and lipid peroxidation substantially. In addition, 6-gingerol reduces inflammation and plays role in the maintenance of colon tissue architecture. Furthermore, the loss of PTEN protein expression was noticed in AOM-treated group, as compared to the 6-gingerol plus AOM treatment group. Based on these findings, it is suggested that colon damage induced by AOM can be prevented by 6-gingerol through its antioxidant potential and anti-inflammatory action. Thus, the consumption of ginger rich in 6-gingerol may have a beneficial effect in treating colon-associated pathogenesis.

## Figures and Tables

**Table 1:** Animal grouping and treatment plan.

Group Number	Group Description	Short Name	Treatment Plan
1	Normal Control	NC	Rats received normal saline solution by oral gavage.
2	Disease control (Negative control)	AOM	Azoxymethane (15 mg/kg b.w.) was administered intraperitoneally in normal saline two times a week [18].
3	6-Gin Treatment + Disease control	6-Gin + AOM	6-Gingerol (50 mg/kg b.w.) was given intraperitoneally two times a week before the administration of AOM (15 mg/kg b.w.).
4	6-Gin treatment only	6-Gin	6-Gingerol (50 mg/kg b.w.) was given intraperitoneally two times a week [19].

NC stands for Normal Control; AOM stands for Azoxymethane; 6-Gin stands for 6-Gingerol.

## Data Availability

The data used to support the findings of this study are included within the article.
